# Supplementary figures and images for: Autophagy Suppresses CCL2 to Preserve Appetite and Prevent Lethal Cachexia
Source: bioRxiv. 2025 Feb 24:2025.02.20.638910. Preprint. [Version 1] doi: 10.1101/2025.02.20.638910 (PMC11888218; doi:10.1101/2025.02.20.638910)

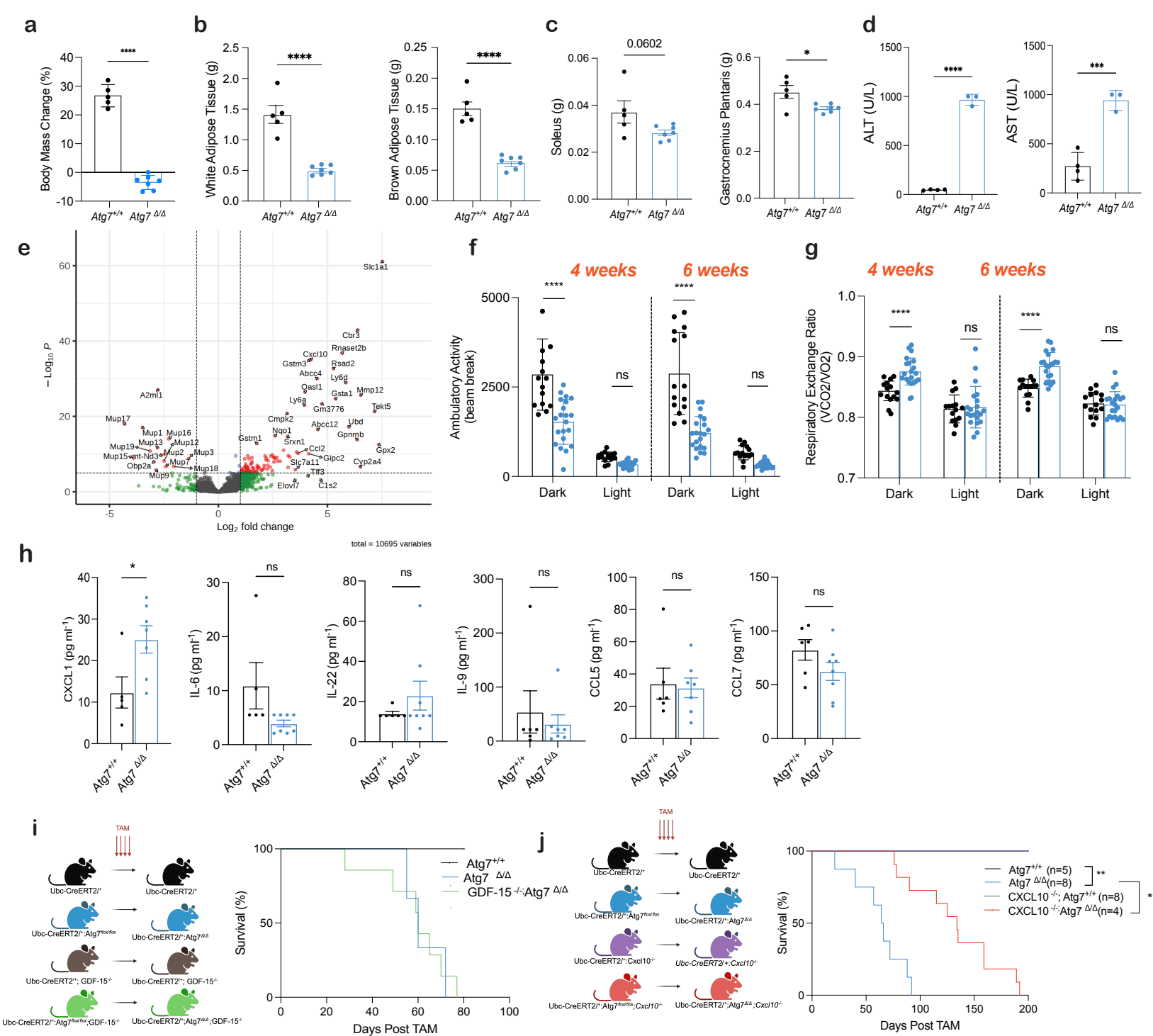

Supplement: Supplement 1 [file media-1.pdf]

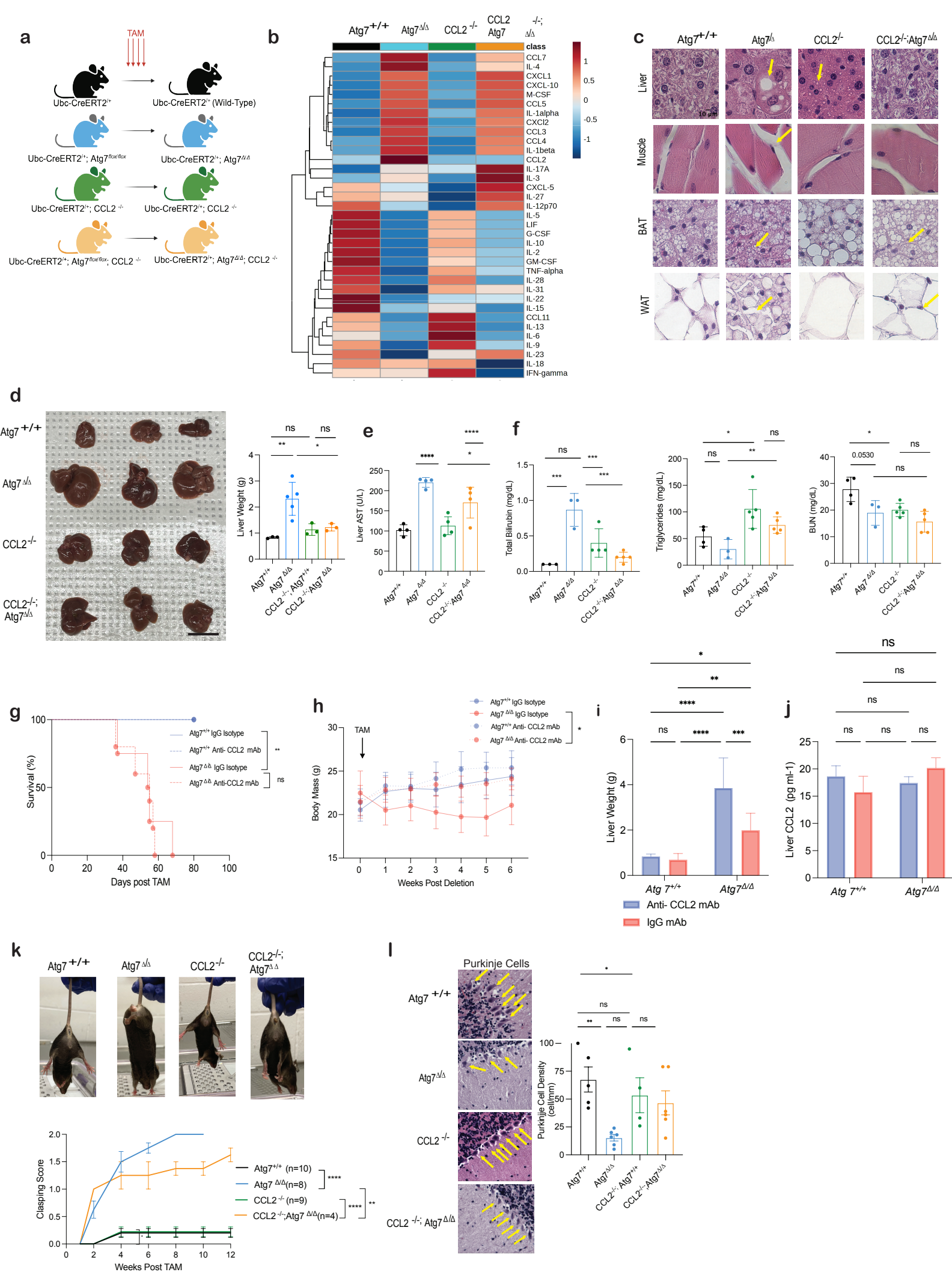

Supplement: Supplement 2 [file media-2.pdf]

**a**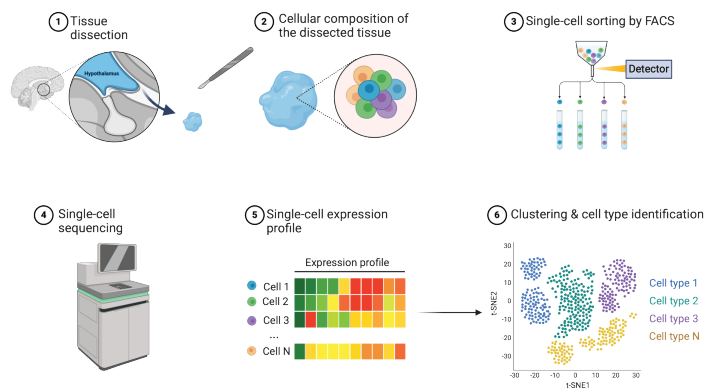**b**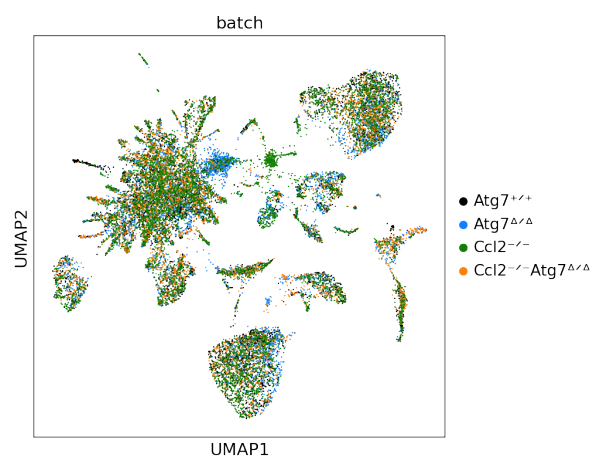**c**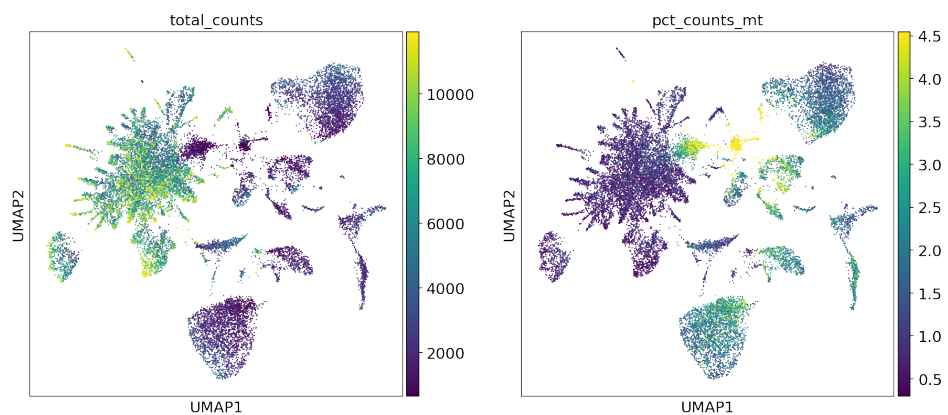**d**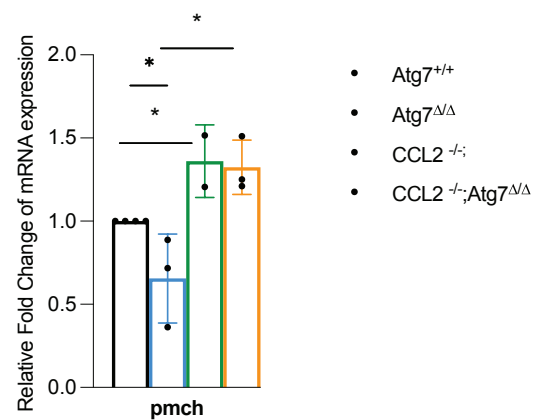

Supplement: Supplement 3 [file media-3.pdf]
